# Wearable gait analysis systems: ready to be used by medical practitioners in geriatric wards?

**DOI:** 10.1007/s41999-022-00629-1

**Published:** 2022-03-03

**Authors:** Malte Ollenschläger, Felix Kluge, Matthias Müller-Schulz, Rupert Püllen, Claudia Möller, Jochen Klucken, Bjoern M. Eskofier

**Affiliations:** 1 Machine Learning and Data Analytics Lab, Friedrich-Alexander-Universität Erlangen-Nürnberg (FAU), Carl-Thiersch-Str. 2b, 91052 Erlangen, Germany; 2 AGAPLESION DIAKONIEKLINIKUM HAMBURG, Hamburg, Germany; 3 AGAPLESION MARKUS KRANKENHAUS, Frankfurt am Main, Germany; 4 AGAPLESION gAG, Frankfurt am Main, Germany; 5 Centre Hospitalier de Luxembourg, Luxembourg Institute of Health, University of Luxembourg, Esch-sur-Alzette, Luxembourg

**Keywords:** Gait, Walking, Geriatric assessment, Instrumentation, Technology transfer

## Abstract

**Purpose:**

We assess feasibility of wearable gait analysis in geriatric wards by testing the effectiveness and acceptance of the system.

**Methods:**

Gait parameters of 83 patients (83.34 ± 5.88 years, 58/25 female/male) were recorded at admission and/or discharge to/from two geriatric inpatient wards. Gait parameters were tested for statistically significant differences between admission and discharge. Walking distance measured by a wearable gait analysis system was correlated with distance assessed by physiotherapists. Examiners rated usability using the system usability scale. Patients reported acceptability on a five-point Likert-scale.

**Results:**

The total distance measures highly correlate (*r* = 0.89). System Usability Scale is above the median threshold of 68, indicating good usability. Majority of patients does not have objections regarding the use of the system. Among other gait parameters, mean heel strike angle changes significantly between admission and discharge.

**Conclusion:**

Wearable gait analysis system is objectively and subjectively usable in a clinical setting and accepted by patients. It offers a reasonably valid assessment of gait parameters and is a feasible way for instrumented gait analysis.

## Key summary points

**Aim:**

To investigate the feasibility of wearable gait analysis in geriatric wards by testing the effectiveness and acceptance of the system.

**Findings:**

Wearable gait analysis can be implemented into geriatric wards, showing its readiness for a transformation from a pure research tool to a practically usable gait analysis system.

**Message:**

Despite good transferability into clinical practice, future research should aim to increase functionality and applicability of wearable gait analysis systems in clinical contexts.

## Introduction

Mobility is an important aspect of healthy aging [1]. An accepted framework for mobility by Webber et al. [2] defines two dimensions of mobility: of life-space locations and mobility determinants. Each life-space, such as home or neighborhood, consists of mobility determinants, such as psychosocial, physical, or environmental. These mobility determinants are inter-dependent. For example, physical limitations may result in decreased environmental mobility and social engagement [2, 3]. In fact, mobility impairments lead to reduced quality of life and dependency in activities of daily living [1, 3, 4]. Furthermore, the effect of decreased mobility extends to the person’s social network, which may lose valuable contributions and need to additionally support this person. Therefore, it is very important for healthy aging, but also for society, to assess and maintain mobility to offer multiple health benefits [1].

One of the variables explaining most variation of different life-spaces is walking speed [5]. Therefore, assessing gait is an important aspect of physical determinants of mobility. In the clinical inpatient context, gait performance is commonly assessed by the Timed-up-and-go (TUG) and the two-minute-walk-test (2MWT) [6, 7]. These measure total time and total distance, respectively. However, detailed gait metrics based on single strides, such as stride time or length, cannot be assessed. Furthermore, gait variability cannot be assessed, as single strides are not regarded. Nonetheless, gait variability is an important parameter, which is related to functional status and fall risk [8–10]. For example, variability of stride length is related to future falls [10–12] and gait speed as well as variability of step time are related to cognitive decline [10, 13]. In turn, detailed information about functional status and potentially about the impact of disease-modifying treatments can be obtained [14–16]. Therefore, the assessment of gait variability is an important part of gait assessment, which can be performed with instrumented gait analysis (IGA), for example, using gait mats or wearable sensors [10, 17].

Wearable systems are predicted to change healthcare delivery and to improve medical treatments as well as patient monitoring [18]. For example, they are a promising approach for fall prevention during hospital stays [19]. Advances in IGA in general are reflected by a substantial increase of publications between 2000 and 2020, as well as the work of international consortia in projects, such as FARSEEING or Mobilise-D [20–22]. Although many publications indicate technically valid systems for IGA, transferability to clinical practice is assessed rarely.

A commonly used method for IGA are gait mats, which are accepted as gold standard [23]. A recent analysis by Stuck et al. [24] shows that they are often used for example to assess gait speed. One of the most popular systems is GAITRite [25]. Implementing it in clinics has been found to be feasible [26]. However, gait mats have a limited capture volume, are not portable, and cannot assess gait parameters during swing phase. In addition, using a gait mat results in a distortion of the assessed gait speed. While older patients without mobility impairment walk significantly faster on the gait mat, older patients with mobility impairment walk significantly slower on gait mats [24]. Furthermore, 4% of patients refused to walk on the mat in a study of Nocera et al. [26].

A different approach is taken by wearable systems, which employ body worn sensors to assess gait. As wearable gait analysis (WGA) systems can be used ubiquitously, they overcome the previously mentioned drawbacks of gait mats. Furthermore, they are cheaper than other systems, as for example gait mats increasing the cost-efficiency of gait analysis [27, 28].

Wearable sensors can be used to obtain additional parameters of the TUG. The test can automatically be sub-divided into different phases, enabling the measurement of durations for sit-to-stand or turnings [29, 30]. This also allows to assess gait parameters of single strides, such as stride length and in turn gait variability. Regarding the use of WGA systems in clinical settings, Bernhard et al. and Mc Ardle et al. report implementation to be feasible; however, they focus on validity of measured parameters [31, 32]. Acceptance is only assessed implicitly from willingness to participate. Usability for clinical experts is not quantified. Therefore, applicability of WGA to everyday scenarios in geriatric inpatient settings remains unknown.

For a different setting, home environments, low usability and practicality were reported [33, 34]. Keogh et al. [33] assessed usability of wearable sensors in a younger cohort with a mean age of 62 years living independently at home. They found that wearables in general seem to be of low usability and medium acceptance. Ancona et al. [34] conducted a literature review and state that applicability of wearable sensors is limited by practicality. This emphasizes, that systems seem to be developed without taking user perspectives into account and that there is a need to analyze usability.

However, due to the different setting and patient cohort, these results cannot be transferred to geriatric inpatient care. A major difference is that the WGA is used by the patients themselves in home environments, whereas in a geriatric clinical setting the staff would be the user.

We analyze the implementation of a WGA into geriatric inpatient settings. Technical validity of the system has been shown in previous studies [17, 35, 36]. However, it is not clear whether the system can correctly be used by physiotherapists in a clinical setting. For objective analysis regarding the implementation into geriatric inpatient settings, we expect a correlation of walked distance measured with WGA and annotated by physiotherapists. Furthermore, we expect significant differences in gait parameters between admission and discharge [37]. For subjective analysis, we evaluate usability and acceptance of the system from physiotherapists’ and patients’ perspective, respectively.

The results of this study show that WGA can be implemented into geriatric wards, showing its readiness for a transformation from a pure research tool to a practically usable gait analysis system. Thus, WGA might be used to help healthcare institutions to cope with changing incentives that emerge from a value-based healthcare transformation in future.

## Methods

### Gait analysis system

**Fig. 1:**
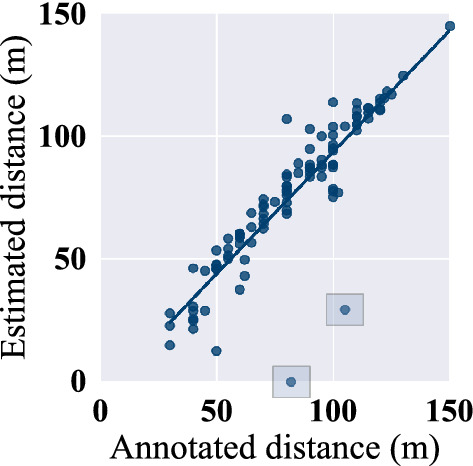
Wearable gait analysis system consisting of a tablet, two inertial measurement units which can be attached to the shoe’s instep [38]

The WGA used in this study comprises of orthopedic shoes, two inertial measurement units (IMUs), and a tablet for interaction with the system [38]. The grey IMUs are attached to the shoes’ instep for recording gait parameters (Fig. 1). They measure acceleration and gyroscopic rate signals, which are transferred to the tablet wirelessly. Spatiotemporal gait parameters are calculated from those quantities as follows: First, a walking trial is segmented into single strides. Then, specific gait events such as initial and final foot contact are detected. Subsequently, the foot’s spatial three-dimensional trajectory is reconstructed. From this, the system reports fourteen spatiotemporal gait parameters, such as stride length and heel strike angle (Table 1).

**Table 1:** Gait parameters reported by the WGA used in this study (mobile GaitLab)

Gait parameter (unit)	Description
Arc length (m)	Path length in transversal plane (floor level) between two consecutive minimum velocity events
Heel strike angle (deg)	Angle between foot sole and floor at heel-strike event
Landing impact intensity (g)	Measured acceleration at detected heel-strike event
Max. lateral excursion (m)	Maximum position along medio-lateral axis relative to position at previous minimum-velocity event
Max. toe clearance (m)	Maximum lift of toe during swing phase
Stance time (s)	Time between heel-strike and toe-off
Stance time (%)	Stance time (s) relative to stride time (s)
Stride length (m)	Shortest distance between to minimum velocity events
Stride time (s)	Time between two consecutive heel-strike events
Speed (m/s)	Stride length divided by stride time
Swing time (s)	Time between toe-off and heel-strike
Swing time (%)	Swing time relative to stride time (s)
Toe off angle (deg)	Angle between foot sole and floor at toe-off event
Turning angle (deg)	Angle of rotation in transversal plane between to consecutive minimum velocity events

The algorithm has previously been technically validated [17, 35, 36]. System validity has been assessed in comparison with GAITRite and a camera-based system. Stride length can be assessed with an error of less than two centimeters [17, 36]. The system shows an excellent test–retest reliability with intraclass correlation > 0.81 [36].

We implemented the use of this system into standardized assessments at admission and discharge of two geriatric inpatient clinics of AGAPLESION gAG in Germany. For organizational reasons, gait assessment took place 1 day after/before admission/discharge. Physiotherapists were trained by a medical engineer in using the system during a 20-min group session. A total of 11 physiotherapists used the system and two physiotherapists per clinic were key users. They were accompanied by the medical engineer during exemplary gait assessments with patients after the group session. The physiotherapists were responsible for handling the sensor system, such that the patients did not need to interact with the system before, during or after the gait assessment. For gait assessment, two of the commonly used standardized gait tests were selected: TUG [6] and 2MWT [7] along a 60 m hallway. For the TUG, patients were instructed to walk at a comfortable and safe space [6]. Instructions regarding the 2MWT were given as suggested by American Thoracic Society [39]. Accordingly, patients were allowed to rest during the test, if necessary.

### Data set

The total covered distance was calculated by summing up the estimated stride lengths of the left and right foot separately and subsequent averaging of both estimates. In addition, physiotherapists annotated total walked distance during 2MWT using 10-m marks on the floor as reference. If the patient stopped between two 10-m marks, the physiotherapist estimated the additional distance in meters. All patients were included in a cross-sectional analysis. Patients that were recorded at admission and discharge were additionally included in longitudinal analysis.

Eight physiotherapists from two clinics answered the system usability scale (SUS) [40] 4 months and 1 year after delivery of the system. During the 12-month period, the physiotherapists had used the WGA system 84 times. Four physiotherapists answered the SUS on both occasions and four at one of the occasions.

Acceptability was rated by 25 patients, asking for their agreement to the following sentence: “The conducted sensor-based gait analysis is acceptable”. A five-point Likert scale with items ‘strongly disagree’, ‘disagree’, ‘partly agree/partly disagree’, ‘agree’, and ‘strongly agree’ was used.

### Statistical analysis

During the hospital stay, patients received individual therapy. Among others, this included gait training as part of physiotherapy. Therefore, we expected an improvement of gait parameters in the longitudinal analysis. For example, stride length is expected to increase and stride time is expected to decrease between admission and discharge [37]. To verify the expectation that gait parameters differ between admission and discharge, we tested the null-hypothesis “gait parameters do not differ between admission and discharge”. We tested for normality using the Shapiro–Wilk test (Table 2). Subsequently, we used a two-sided paired t-test, or the Wilcoxon signed-rank test, if gait parameters either at admission or discharge were non-normally distributed. Analysis was performed using Python’s scipy package, version 1.5.0. [41]. In addition, we calculated the effect size using Cohen’s d [42].

**Table 2:** Gait parameters recorded using the WGA (mobile GaitLab). Non-Normality (p-norm) tested using Shapiro–Wilk-Test. Non-difference (p-diff) between admission and discharge tested using Wilcoxon or *t* test

Gait parameter	Admission	Discharge	Difference	*p*-diff	Effect size
Stride length (m)	0.92 (0.21)	0.98 (0.20)	0.06 (0.12)	0.002	0.27
Stride time (s)	1.38 (0.26)	1.31 (0.20)	− 0.07 (0.16)	0.003	0.31
Speed (m/s)	0.70 (0.24)	0.78 (0.24)	0.08 (0.14)	< 0.001	0.31
Heel strike angle (deg)	8.16 (4.35)	9.14 (4.34)	0.98 (2.54)	0.01	0.23
TUG time (s)	24.70 (24.70)	20.71 (16.79)	− 3.99 (16.97)	< 0.001	0.28
Two-minute walk distance (m)	76.81 (29.39)	84.41 (30.46)	7.60 (22.31)	0.003	0.25

Values reported as mean (standard deviation). *N* = 48

### Patient characteristics

Between June 2018 and June 2020, 83 patients were assessed in 125 sessions using the WGA. Patient age (range, mean, median) was (68–95, 83.3, 83.0) years and the sex ratio (f/m) was 58/25. They stayed in the hospital for 17 ± 7 days (mean ± standard deviation). For a subset of those patients two gait assessments were performed, and therefore, they were included in the longitudinal analysis. They had an age of (68–95, 82.1, 80.5) and the sex ratio was 31/17. Age of patients asked for acceptability was (71–93, 82.6, 83.0) years and the sex ratio was 12/13.

The most frequently used walking aid was a wheeled walker, as shown in Table 3. It was used in 56% of the gait assessments. Another 12% did not use walking aids. For 20%, no documentation regarding walking aids was made. The most frequent diagnoses were fracture of femur and abnormalities of gait and mobility (28% of patients). Functional status was assessed using the Barthel Index (BI). Patients included in the cross-sectional analysis had an average BI of 59. For 46 patients of the longitudinal analysis, BI was available at admission and discharge. They had a BI of 48 ± 11 at admission and 72 ± 15 at discharge, corresponding to an average improvement of 24 during the hospital stay.

**Table 3:** Patient characteristics for patients included in the cross-sectional analysis (*N* = 83)

Sex (f/m)	58/25
Age (y)	83.34 ± 5.88
Days in hospital (d)	17 ± 7
Barthel-Index	59 ± 17
Three most frequent diagnoses (*N* patients)	
S72: Fracture of femur	13
R26: Abnormalities of gait and mobility	10
I35: Aortic (valve) stenosis	4
Walking aids (percentage of gait assessments)	
Wheeled walker	56%
No walking aid	12%
Walking stick	5%
Crutches	5%
Walker	2%
No remark	20%

This study was conducted at an acute geriatrics ward in Frankfurt, Germany and a mixed acute geriatrics/early rehabilitation ward in Hamburg, Germany. It was approved by the local ethics committees: Nr. 3081, 21.02.2019, IRB, state chamber of physicians, Frankfurt am Main, Germany; Nr. 722, 19.08.2019, IRB, state chamber of physicians, Hamburg, Germany. Patients were eligible if they were regularly admitted to a geriatric inpatient ward, were able to walk, and gave informed consent according to the Declaration of Helsinki. Exclusion criteria were infections or wounds at the feet or other contraindications as determined by the physiotherapist or physician, such as inability to walk for 2 min. In addition, patients with severe cognitive impairments and demented patients were not included due to ethical considerations regarding the ability to consent.

## Results

**Fig. 2:**
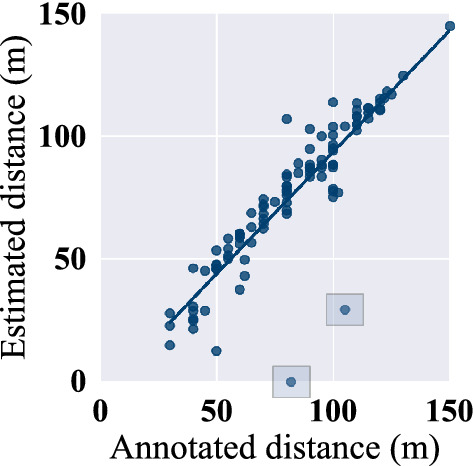
Correlation of measured distance in 2MWT with manual annotation (pearson *r* = 0.89, *p* < 0.001)

For cross-sectional analysis, we compared the covered distance within 2MWT as annotated by physiotherapists with the distance estimated by the WGA. They highly correlate, yielding a Pearson correlation coefficient of 0.89 (Fig. 2). In two of the 125 sessions, the automatic distance calculation did not work as expected. In one session, no strides were detected. Further analysis revealed that one sensor was inserted upside down into the shoe. In a second session, the gait analysis system correctly detected 80 strides for the left foot but only 21 strides for the right foot. In the same session, mean maximum toe clearance was 4.37 cm for the left foot but only 1.47 cm for the right foot.

For longitudinal analysis, we compared five gait parameters at admission with those at discharge. Significant differences were found for stride length (+ 6 cm), stride time (− 0.07 s), TUG time (− 3.99 s), gait speed (+ 0.07 m/s) and heel strike angle (1°) (Table 2).

Physiotherapists rated the system with an average score of 81.67 after 4 months. The survey was combined with an informal interview, which was used to obtain suggestions for a potential improvement of the system. Although this led to several improvements of the user interface, the SUS score was lower after 1 year (72.91). The most prominently reported reason for the lower rating by physiotherapists were issues regarding the connectivity between sensors and tablet. Acceptability was rated positively by patients. In detail, 17 of 25 patients strongly agreed, seven patients agreed, and one patient partly agreed and partly disagreed with the statement that the system is acceptable.

## Discussion

Interest in WGA has increased in the last two decades. Their portability and low-cost offer a promising alternative to other systems based on optical motion capture or gait mats [27, 28]. However, translational research about implementing such systems into clinical workflows of geriatric wards is missing. To the best of our knowledge, this is the first study, which investigates usability and acceptability of assessing gait parameters in geriatric wards. We implemented a WGA into the clinical practice of two geriatric wards. We showed good usability, better acceptance than in a previous study using a gait mat [26], and that obtained gait parameters are reasonable [37].

Regarding cross-sectional analysis, the covered distances during 2MWTs was correctly assessed. Only for two sessions, there was an obvious underestimation, potentially detectable using simple algorithms. In one of those sessions, detected strides of the right foot had a low clearance according to the sensor system, which might indicate shuffling gait. The currently used algorithm is not able to algorithmically evaluate shuffling gait, which might be the reason for the large number of undetected strides. For this reason, it makes sense to evaluate other stride detection algorithms, as, for example, Hidden Markov Models [43]. Furthermore, a quantification of shuffling gait or foot drag might add value to the analysis.

For the longitudinal analysis, all listed gait parameters showed a significant change (*p* < 0.05), which is in accordance with the functional improvement regarding the BI. The direction of change is in line with those reported in literature [37, 44]. Furthermore, the magnitude of change is in agreement with the results of Schwenk et al. [37], who investigated gait parameters of geriatric inpatients using a gait mat. They found an increase of seven centimeters in stride length for patients using a walking aid, which is the same as in our study. In both studies, stride time decreased by 0.07 s. The TUG duration decreased by 4.34 s in the study of Schwenk et al. and by 3.99 s in our study. Contrary to Schwenk et al. who reported a gait speed increase of 0.13 m/s, we observed an increase of only 0.07 m/s in. This might be attributed to the fact that the patients walked only approximately 5 m in the study of Schwenk et al., but for 2 min in our study resulting in a mean distance of 82 m. Due to the longer distance, patients may have fatigued over time.

Expected gait parameters from literature were reproduced in our study, suggesting that gait parameters were assessed correctly. Thus, we show that physiotherapists without technical expertise can accurately assess a manifold of gait parameters by employing a WGA.

In addition, we observed significant differences in heel-strike angle between admission and discharge. To the best of our knowledge, this has previously not been reported but complements results from previous studies, which found differences of heel strike angle in first-time versus frequent wheeled walker users as well as a relation of heel strike angle with frailty [45, 46]. Our study gives supporting evidence that measuring the foot’s orientation during the gait cycle might be of value in longitudinal analysis. This further advocates for the use of WGAs instead of gait mats, which cannot assess the heel strike angle or foot clearance.

Results of the SUS indicate good usability of the WGA, since the score is above 68 [47]. Improvements can still be achieved, mainly regarding the connectivity of sensors and tablet.

Since transfer of gait parameters from the tablet to computer systems was performed digitally, we avoided errors due to manual data transfer. In comparison, Nocera et al. [26], who used manual data copying, report an error rate of 13%. In our study, 68% of patients included in this analysis strongly agreed that the use of the WGA system is acceptable, whereas in the study of Nocera and colleagues, 62% strongly agreed. In contrast to studies regarding multimodal sensing or wearable sensing in geriatric home environments, this rate of acceptance seems to be low. For those scenarios, acceptance rates after use of the system of 91% [48] and 93% [49] have been reported. However, these studies refer to home-monitoring and thus a different scenario. In the same way as our study, they may have been subject to a selection bias. Furthermore, in one study, it is not clear how acceptance was assessed [49], and in the other study, the employed scale had three items in contrast to five items in our study [48]. Taking into account, that the most negative answer regarding acceptability in our study was ‘partly agree, partly disagree’, we argue that our results are actually in the same range as those reported in the previously mentioned studies.

Considering these overall positive results, WGAs can be successfully implemented in geriatric inpatient settings. We support this assumption by showing that gait parameters can be obtained correctly by physiotherapists without technical expertise. Our results provide evidence that measuring heel strike angle, and, therefore, using WGAs instead of gait mats, can yield valuable information.

A limitation of our study is that not all patients who were able to walk for 2 min participated in the study. This is due to the documentation overload introduced by obtaining informed consent and leading to a lack of time. Furthermore, a possible selection bias may have led to inclusion of patients who are likely to participate in the study. Thus, the assessment of acceptability may be positively biased. In addition, the amount of information obtained from answering the Likert scale is limited. Therefore, these results need to be interpreted cautiously. However, the difference regarding the assessment of patients included in the study and other regularly assessed patients was small. The only difference for patients included in the study in contrast to other regularly assessed patients was to wear different shoes with a sensor attached. Since the system was handled by the physiotherapists, most important barriers to technology acceptance in older people, namely low usability and interface complexity are avoided in this study [50]. In addition, a selection bias may have resulted in not including patients with mobility disorders, potentially impacting the distribution of assessed gait parameters. However, 48% of the included patients were diagnosed with fracture of femur or abnormalities of gait and mobility, which shows that patients with mobility impairments were included. Furthermore, the literature shows comparable gait parameters to those we assessed, as discussed above. WGA systems can be used in clinical practice. Despite the overall positive results indicating good transferability of WGA into clinical practice, the mere assessment of objective gait parameters does not have an inherent value for practitioners. As stated by Routhier et al., there is still a need for increased functionality as well as applicability [51]. It would be necessary to further process the gait parameters to generate meaningful results for clinical practitioners. This could, for example, be solved by generating a clinical interface, which integrates diagnostic information with the results of the gait analysis [27].

By addressing this in future studies, WGA could be used for performance-based gait assessment, including variability measures. This will give medical practitioners more detailed, objective, and reliable feedback to assess patients’ mobility.

## Declarations

### Institutions of data recording

AGAPLESION DIAKONIEKLINIKUM HAMBURG, Hamburg, Germany and AGAPLESION MARKUS KRANKENHAUS, Frankfurt am Main, Germany.

### Institution of data analysis

Machine Learning and Data Analytics Lab, Friedrich-Alexander-Universität Erlangen-Nürnberg (FAU), Erlangen, Germany.
