# Correction to: Wearable gait analysis systems: ready to be used by medical practitioners in geriatric wards?

**DOI:** 10.1007/s41999-022-00646-0

**Published:** 2022-04-12

**Authors:** Malte Ollenschläger, Felix Kluge, Matthias Müller-Schulz, Rupert Püllen, Claudia Möller, Jochen Klucken, Bjoern M. Eskofier

**Affiliations:** 1 Machine Learning and Data Analytics Lab, Friedrich-Alexander-Universität Erlangen-Nürnberg (FAU), Carl-Thiersch-Str. 2b, 91052 Erlangen, Germany; 2 AGAPLESION DIAKONIEKLINIKUM HAMBURG, Hamburg, Germany; 3 AGAPLESION MARKUS KRANKENHAUS, Frankfurt am Main, Germany; 4 AGAPLESION gAG, Frankfurt am Main, Germany; 5 Centre Hospitalier de Luxembourg, Luxembourg Institute of Health, University of Luxembourg, Esch-sur-Alzette, Luxembourg

## Correction to: European Geriatric Medicine 10.1007/s41999-022-00629-1

In this article the graphics relating to Figs. 1 and 2 captions had been interchanged; the figures should have appeared as shown below. The original article has been corrected.

**Fig. 1:**
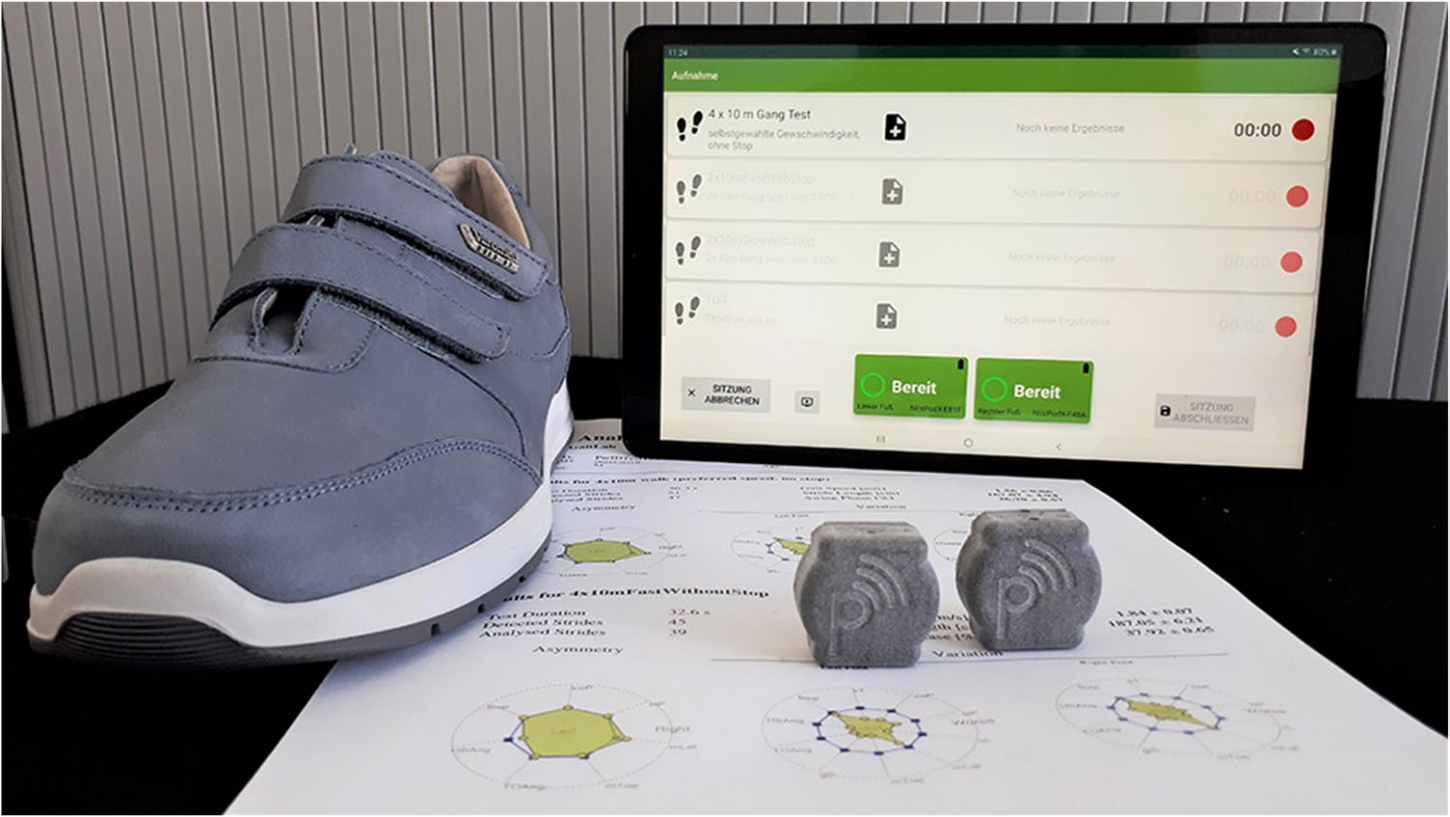
Wearable gait analysis system consisting of a tablet, two inertial measurement units which can be attached to the shoe’s instep [38]

**Fig. 2:**
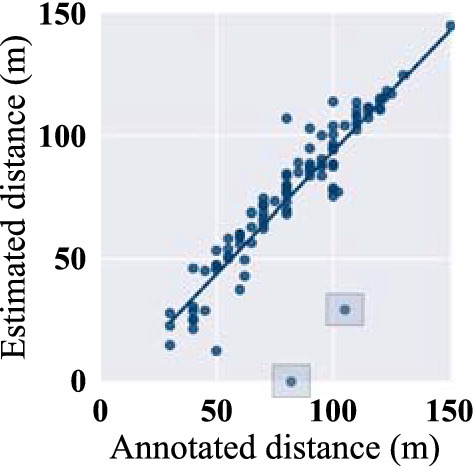
Correlation of measured distance in 2MWT with manual annotation (pearson *r* = 0.89, *p* < 0.001)

